# Stress Induced Neuroplasticity and Mental Disorders 2018

**DOI:** 10.1155/2018/5382537

**Published:** 2018-08-16

**Authors:** Fushun Wang, Fang Pan, Lee A. Shapiro, Jason H. Huang

**Affiliations:** ^1^Institute of Emotional Studies, School of Psychology, Nanjing University of Chinese Medicine, Nanjing 210023, China; ^2^Department of Neurosurgery, Baylor Scott & White Health, Temple, TX 76508, USA; ^3^Department of Medical Psychology, School of Basic Medical Sciences, Shandong University, Jinan 250012, China; ^4^Department of Surgery, Texas A&M College of Medicine, Temple, TX 76504, USA; ^5^Texas A&M University College of Medicine, Temple, TX 76508, USA

Stress was first named as the general adaptation syndrome to define a process when the body confronts noxious agents. Nearly a century has passed since Hans Selye first introduced the word stress in 1936, and this brilliant idea about stress has helped an entirely new field to be forged and attracted thousands of researchers to work on the biological mechanism of stress. Now stress is extended to the field of psychology and redefined as the presence of acute or persistent physiological or psychological threats to the organism that result in significant strain on the body's compensatory systems.

In face of stress, the first reaction of the body is an alarm reaction, preparing the body to “fight or flight,” as was motivated by the emotional changes, such as fear (flight) and anger (fight) [1, 2]. Nowadays, it is found that emotional stress affects the organism even more seriously, and most of the mental diseases are due to stress-induced emotional changes, which can induce significant strains to the body. Folkman et al. proposed that there are two kinds of emotional changes [3]: the first is related to threat and fearful emotion, which motivates withdrawal and flight behavior; and the second is related to coping with the situation, when the organism activates energy in the body to cope with the situation and show angry emotions. Thus, *fear and anger are two basic emotions at stressful events*: fear is the scariness at the threat, while anger is trying to cope with the stressful situation [4]. Lazarus proposed that stress depends on cognitive appraisals of situation [5]. He distinguished two kinds of appraisals: the first appraisal is unconscious and fast activating, which is related to harm and threat and induces fearful emotion to motivate avoidance and withdrawal; the second is conscious and concerned with coping with the uncontrolled situation. In the face of threat, the organism was scared at first and showed fearful emotions; then to cope with the threats, the organism collects energy in the body to “fight or flight”. Therefore, fear and anger usually come in a tandem at the stressful events [6]. Fear and anger are hard to be detached and are two sides of the same coin [5].

Lazarus suggested another stage of appraisal after coping with the situation (called reappraisal). At this stage, an individual employs two kinds of reappraisals: problem-focused (cognition), and emotion-focused. If the organism can cope successfully with the stressful situation, the organism will get positive emotions and be happy. If the organism failed to cope with the situation, the organism would get negative emotions and be sad. Therefore, a stressful event-induced emotion will go through “fear-anger-joy or sadness” *emotion flow*. If the organism cannot overcome the stressful events, like the chronic stresses, some mental disorders will appear. The relations of the process with the emotional changes and some mental disorders were shown in our previous editorial. Therefore, when something happens, people will first evaluate whether it is dangerous (fear/anger) or not (calm) and next evaluate if they fit into our need (happy/sad) [7]. This hypothesis is exactly consistent with Lazarus' reappraisal theory about happiness or sadness [5]: the happy and sad emotion are related to the success or failure to cope with stressful situations.

The mechanisms whereby external stressors affecting brain function have been the subject of extensive study over the past half a century. It is well known that the major function of NE is “fight (anger) or flight (fear)” [8, 9]. The stress-induced neural plasticity undoubtedly affects the brain function and may prompt functional alternations in mental disorders. Indeed, stress-induced neuroplasticity plays a critical role in almost all of the mental disorders, and stress has become a synonym for diverse terms of negative emotions, such as depression, anxiety.

In previous special issues, we already collected 9 papers on stress-induced neural plasticity and some neurological diseases. Because of its popularity, we were invited to hold this issue as the annual topic in 2018, and we are glad to get 31 submissions, and 11 of which are accepted for publication. These reviews and experimental papers present more evidence about stress.

In the experimental paper “The Neural Basis of Fear Promotes Anger and Sadness Counteracts Anger,” J. Zhan et al. probed into the relationships about basic emotions and reported that fear leads to anger. They tested this hypothesis with MRI and found that the selective involvement of different brain regions in different basic emotions might be the reason for the relationship between the basic emotions. For example, they found that the posterior insula (PI) is involved in sadness, while the anterior insula (AI) is involved in fear. Their interesting data helped explain the relationship between the basic emotions: fear-anger-sadness.

In the review paper “Persistent Stress-Induced Neuroplastic Changes in the Locus Coeruleus/Norepinephrine System,” O. Borodovitsyna et al. reviewed papers about neural mechanisms underlying stress, especially locus coeruleus/norepinephrine system. In this paper, they reported how stress changes the structure and function of LC from a genetic, cellular, and neuronal circuitry/transmission perspective. They further linked stress to altered LC function and pathogenesis of posttraumatic stress disorder.

Chronic stress often induces neural plasticity in the brain at molecular, cellular level. In the experimental paper “Recovery of Chronic Stress-Triggered Changes of Hippocampal Glutamatergic Transmission,” M. Lin et al. probed into the dynamic changes in excitatory transmission in the hippocampus and investigated the spontaneous recovery of spatial memory function and glutamatergic transmission in the hippocampus after chronic stress. They found that chronic unpredicted mild stress transiently increased AMPA receptor GluA2/3 subunit expression, together with elevated PICK-1 protein expression. They further probed into the spontaneous recovery after the stress is removed.

In the experimental paper “Metabolic Changes Associated with a Rat Model of Diabetic Depression Detected by Ex Vivo 1H Nuclear Magnetic Resonance Spectroscopy in the Prefrontal Cortex, Hippocampus, and Hypothalamus,” K. Liu et al. reported stress-induced depression in diabetic depression. They used magnetic resonance spectroscopy and immunohistochemistry to investigate the metabolic and pathological changes in the rat brain and found that the levels of glutamate decrease at depression, which are possibly due to dysfunction of neurons and astrocytes.

In the experimental paper “Context and Time Matter: Effects of Emotion and Motivation on Episodic Memory Overtime,” Q. Sun et al. compared the reaction times about reward stimuli, punishment stimuli, and stressful stimuli in human subjects and found that stressful stimuli are highly arousing and can trigger more efficient memory consolidation.

In the experimental paper “Direct Electrophysiological Mapping of Shape-Induced Affective Perception,” Y. Li et al. reported a very interesting behavior and ERP study about emotional arousal of pleasant and unpleasant stimuli. Consistent with Lazarus's theory of two processing pathways of the brain about stressful stimuli and hedonic stimuli, they found that stressful stimuli, such as angry face, can induce faster and larger response in the earlier ERP responses, particular P1, N1.

In the experimental paper “TLR4-NF-*ĸ*B Signal Involved in Depressive-Like Behaviors and Cytokine Expression of Frontal Cortex and Hippocampus in Stressed C57BL/6 and ob/ob Mice,” Y. Wang et al. reported that elevated levels of cytokines such as interleukin- (IL-) 1*β*, IL-6, and tumor necrosis factor-*α* (TNF-*α*) are closely associated with pathology of depression in obesity mice during stress processing.

In the experimental paper “Danshen-Honghua Ameliorates Stress-Induced Menopausal Depression in Rats,” S. Gu et al. reported neurotransmitter and sex hormone changes in an animal model of menopause depression. In addition, they found that a kind of Chinese herb can help treat menopause depression. This paper will help understand the pathogenesis of perimenstrual depression.

In the experimental paper “Language and Sensory Neural Plasticity in the Superior Temporal Cortex of the Deaf,” M. Que et al. showed plausible neural pathways for auditory reorganization for deaf patients. They probed into the correlations of activations of the reorganized cortical areas with developmental factors and provided unique evidence towards the understanding of neural circuits involved in cross-modal plasticity.

In the experimental paper “Examination Stress Results in Attentional Bias and Altered Neural Reactivity in Test-Anxious Individuals,” X. Zhang et al. studied the test stress in college students, using ERP (event-related potentials). They found that test stress can induce functional perturbations of brain circuitry that reacts rapidly to test threat.

In the experimental paper “Relationship between Insulin Levels and Nonpsychotic Dementia: A Systematic Review and Meta-Analysis,” Q. Pan et al. reported a special relationship about stress with dementia in human subjects. They found that dementia is related with insulin, which can induce a variety of neural plasticities, such as apoptosis in neurons, thus cognitive functions, such as learning and memory.

Collectively, these studies demonstrate that stress can induce many critical changes in many mental disorders. We hope that this special issue will stimulate interests in the field of the mechanism of stress inducing the neural plasticity and will help achieve a deeper understanding of the molecular mechanism of stress-induced mental disorders.

*Fushun Wang* *Fang Pan* *Lee A. Shapiro* *Jason H. Huang*
